# Towards generalisable hate speech detection: a review on obstacles and solutions

**DOI:** 10.7717/peerj-cs.598

**Published:** 2021-06-17

**Authors:** Wenjie Yin, Arkaitz Zubiaga

**Affiliations:** School of Electronic Engineering and Computer Science, Queen Mary University of London, London, United Kingdom

**Keywords:** Hate speech, Text classification, Abusive language, Social media, Literature review, Generalisation

## Abstract

Hate speech is one type of harmful online content which directly attacks or promotes hate towards a group or an individual member based on their actual or perceived aspects of identity, such as ethnicity, religion, and sexual orientation. With online hate speech on the rise, its automatic detection as a natural language processing task is gaining increasing interest. However, it is only recently that it has been shown that existing models generalise poorly to unseen data. This survey paper attempts to summarise how generalisable existing hate speech detection models are and the reasons why hate speech models struggle to generalise, sums up existing attempts at addressing the main obstacles, and then proposes directions of future research to improve generalisation in hate speech detection.

## Introduction

The Internet saw a growing body of user-generated content as social media platforms flourished (Schmidt & Wiegand, 2017; Chung et al., 2019). While social media provides a platform for all users to freely express themselves, offensive and harmful contents are not rare and can severely impact user experience and even the civility of a community (Nobata et al., 2016). One type of such harmful content is **hate speech**, which is speech that **directly attacks** or **promotes hate** towards a group or an individual member based on their actual or perceived aspects of **identity**, such as ethnicity, religion, and sexual orientation (Waseem & Hovy, 2016; Davidson et al., 2017; Founta et al., 2018; Sharma, Agrawal & Shrivastava, 2018). Major social media companies are aware of the harmful nature of hate speech and have policies regarding the moderation of such posts. However, the most commonly used mechanisms are very limited. For example, keyword filters can deal with profanity, but not the nuance in the expression of hate (Gao, Kuppersmith & Huang, 2017). Crowd-sourcing methods (e.g., human moderators, user reporting), on the other hand, do not scale up. This means that by the time that a hateful post gets detected and taken down, it has already made negative impacts (Chen, McKeever & Delany, 2019).

The automatic detection of hate speech is thus an urgent and important task. Since the automatic detection of hate speech was formulated as a task in the early 2010s (Warner & Hirschberg, 2012), the field has been constantly growing along the perceived importance of the task.

### Hate speech, offensive language, and abusive language

Although different types of abusive and offensive language are closely related, there are important distinctions to note. Offensive language and abusive language are both used as umbrella terms for harmful content in the context of automatic detection studies. However, while “strongly impolite, rude” and possible use of profanity are seen in the definitions of both (Fortuna & Nunes, 2018), abusive language has a strong component of intentionality (Caselli et al., 2020). Thus, offensive language has a broader scope, and hate speech falls in both categories.

Because of its definition mentioned above, hate speech is also different from other sub-types of offensive language. For example, personal attacks (Wulczyn, Thain & Dixon, 2017) are characterised by being directed at an individual, which is not necessarily motivated by the target’s identity. Hate speech is also different from cyberbullying (Zhao, Zhou & Mao, 2016), which is carried out repeatedly and over time against vulnerable victims that cannot defend themselves.^1^ 1For a more elaborate comparison between similar concepts, see Fortuna & Nunes (2018); Poletto et al. (2020); Banko, MacKeen & Ray (2020)This paper focuses on hate speech and hate speech datasets, although studies that cover both hate speech and other offensive language are also mentioned.

### Generalisation

Most if not all proposed hate speech detection models rely on supervised machine learning methods, where the ultimate purpose is for the model to learn the real relationship between features and predictions through training data, which generalises to previously unobserved inputs (Goodfellow, Bengio & Courville, 2016). The **generalisation performance** of a model measures how well it fulfils this purpose.

To approximate a model’s generalisation performance, it is usually evaluated on a set-aside test set, assuming that the training and test data, and future possible cases come from the same distribution. This is also the main way of evaluating a model’s ability to generalise in the field of hate speech detection.

### Generalisability in hate speech detection

The ultimate purpose of studying automatic hate speech detection is to facilitate the alleviation of the harms brought by online hate speech. To fulfil this purpose, hate speech detection models need to be able to deal with the constant growth and evolution of hate speech, regardless of its form, target, and speaker.

Recent research has raised concerns on the generalisability of existing models (Swamy, Jamatia & Gambäck, 2019). Despite their impressive performance on their respective test sets, the performance significantly dropped when the models are applied to a different hate speech dataset. This means that the assumption that test data of existing datasets represent the distribution of future cases is not true, and that the generalisation performance of existing models have been severely overestimated (Arango, Prez & Poblete, 2020). This lack of generalisability undermines the practical value of these hate speech detection models.

So far, existing research has mainly focused on demonstrating the lack of generalisability (Gröndahl et al., 2018; Swamy, Jamatia & Gambäck, 2019; Wiegand, Ruppenhofer & Kleinbauer, 2019; Fortuna, Soler-Company & Wanner, 2021), apart from a handful of studies that made individual attempts at addressing aspects of it (Karan & Šnajder, 2018; Waseem, Thorne & Bingel, 2018; Arango, Prez & Poblete, 2020). Recent survey papers on hate speech and abusive language detection (Schmidt & Wiegand, 2017; Fortuna & Nunes, 2018; Al-Hassan & Al-Dossari, 2019; Mishra, Yannakoudakis & Shutova, 2019; Vidgen et al., 2019; Poletto et al., 2020; Vidgen & Derczynski, 2020) have focused on the general trends in this field, mainly by comparing features, algorithms and datasets. Among these, Fortuna & Nunes (2018) provided an in-depth review of definitions, Vidgen et al. (2019) concisely summarised various challenges for the detection of abusive language in general, Poletto et al. (2020) and Vidgen & Derczynski (2020) created extensive lists of resources and benchmark corpora while Al-Hassan & Al-Dossari (2019) focused on the special case of the Arabic language.

This survey paper thus contributes to the literature by providing (1) a comparative summary of existing research that demonstrated the lack of generalisability in hate speech detection models, (2) a systematic analysis of the main obstacles to generalisable hate speech detection and existing attempts to address them, and (3) suggestions for future research to address these obstacles.

This paper is most relevant to any researcher building datasets of, or models to detect, online hate speech, but can also be of use for those who work on other types of abusive or offensive language.

## Survey Methodology

For each of the three aims of this paper mentioned above, literature search was divided into stages.

### Sources of search

Across different stages, Google Scholar was the main search engine, and two main sets of keywords were used. References and citations were checked back-and-forth, with the number of iterations depending on how coarse or fine-grained the search of that stage was.

• General keywords: “hate speech”, “offensive”, “abusive”, “toxic”, “detection”, “classification”.
• Generalisation-related keywords: “generalisation” (“generalization”), “generalisability” (“generalizability”), “cross-dataset”, “cross-domain”, “bias”.

We started with a pre-defined set of keywords. Then, titles of proceedings of the most relevant recent conferences and workshops (Workshop on Abusive Language Online, Workshop on Online Abuse and Harms) were skimmed, to refine the set of keywords. We also modified the keywords during the search stages as we encountered new phrasing of the terms. The above keywords shown are the final keywords.

### Main literature search stages

Before starting to address the aims of this paper, an initial coarse literature search involved searching for the general keywords, skimming the titles and abstracts. During this stage, peer-reviewed papers with high number of citations, published in high-impact venues were prioritised. Existing survey papers on hate speech and abusive language detection (Schmidt & Wiegand, 2017; Fortuna & Nunes, 2018; Al-Hassan & Al-Dossari, 2019; Mishra, Yannakoudakis & Shutova, 2019; Vidgen et al., 2019; Poletto et al., 2020; Vidgen & Derczynski, 2020) were also used as seed papers. The purpose of this stage was to establish a comprehensive high-level view of the current state of hate speech detection and closely related fields.

For the first aim of this paper—building a comparative summary of existing research on generalisability in hate speech detection—the search mainly involved different combinations of the general and generalisation-related keywords. As research on this topic is sparse, during this stage, all papers found and deemed relevant were included.

Building upon the first two stages, the main obstacles towards generalisable hate speech detection were then summarised: (1) presence of non-standard grammar and vocabulary, (2) paucity of and biases in datasets, and (3) implicit expressions of hate. This was done through extracting and analysing the error analysis of experimental studies found in the first stage, and comparing the results and discussions of the studies found in the second stage. Then, for each category of obstacles identified, another search was carried out, involving combinations of the description and paraphrases of the challenges and the general keywords. The search in this stage is the most fine-grained, in order to ensure coverage of both the obstacles and existing attempts to address them. After the main search stages, the structure of the main findings in the literature was laid out. During writing, for each type of findings, the most representative studies were included in the writing up. We defined the relative representativeness within studies we have found, based on novelty, experiment design and error analysis, publishing venues, and influence. We also prioritised studies that addressed problems specific to hate speech, compared to better-known problems that are shared with other offensive language and social media tasks.

## Generalisation Studies in Hate Speech Detection

Testing a model on a different dataset from the one which it was trained on is one way to more realistically estimate models’ generalisability (Wiegand, Ruppenhofer & Kleinbauer, 2019). This evaluation method is called cross-dataset testing (Swamy, Jamatia & Gambäck, 2019) or cross-application (Gröndahl et al., 2018), and sometimes cross-domain classification (Wiegand, Ruppenhofer & Kleinbauer, 2019) or detection (Karan & Šnajder, 2018) if datasets of other forms of offensive language are also included.

As more hate speech and offensive language datasets emerged, a number of studies have touched upon cross-dataset generalisation since 2018, either studying generalisability per se, or as part of their dataset validation. The datasets they use (Table 1) to some extent reflect the best-known datasets in hate speech and other types of offensive language. These studies are further compared in Table 2 in terms of the models and datasets they used. As different datasets and models were investigated, instead of specific performance metrics, the remainder of this section will discuss the general findings of these studies, which can be roughly grouped into those on models and those on training and evaluation data.

**Table 1:** English datasets used in cross-dataset generalisation studies. Positive labels are listed with their original wording. Expert annotation type include authors and experts in social science and related fields. ?: Type of annotations not available in original paper, the found descriptions are thus included. Note that only datasets used in generalisation studies are listed—for comprehensive lists of hate speech datasets, see Vidgen & Derczynski (2020) and Poletto et al. (2020).

Dataset name	Publication	Source	Positive labels	Annotator type
Waseem	Waseem & Hovy (2016) Waseem (2016)	Twitter	Racism Sexism	Expert; Expert and crowdsourcing
Davidson	Davidson et al. (2017)	Twitter	Hate speech Offensive	Crowdsourcing
Founta	Founta et al. (2018)	Twitter	Hate speech Offensive	Crowdsourcing
HatEval	Basile et al. (2019)	Twitter	Hateful	Expert and crowdsourcing
Kaggle	Jigsaw (2018)	Wikipedia	Toxic Severe toxic Obscene Threat Insult Identity hate	Crowdsourcing
Gao	Gao & Huang (2017)	Fox News	Hateful	? (Native speakers)
AMI	Fersini, Nozza & Rosso (2018) Fersini, Rosso & Anzovino (2018)	Twitter	Misogynous	Expert
Warner	Warner & Hirschberg (2012)	Yahoo! American Jewish Congress	Anti-semitic Anti-black Anti-asian Anti-woman Anti-muslim Anti-immigrant Other-hate	? (Volunteer)
Zhang	Zhang, Robinson & Tepper (2018)	Twitter	Hate	Expert
Stromfront	De Gibert et al. (2018)	Stormfront	Hate	Expert
Kumar	Kumar et al. (2018b)	Facebook, Twitter	Overtly aggressive Covertly aggressive	Expert
Wulczyn	Wulczyn, Thain & Dixon (2017)	Wikipedia	Attacking	Crowdsourcing
OLID (OffensEval)	Zampieri et al. (2019a)	Twitter	Offensive	Crowdsourcing
AbuseEval	Caselli et al. (2020)	Twitter	Explicit (abuse) Implicit (abuse)	Expert
Kolhatkar	Kolhatkar et al. (2019)	The Globe and Mail	Very toxic Toxic Mildly toxic	Crowdsourcing
Razavi	Razavi et al. (2010)	Natural Semantic Module Usenet	Flame	Expert
Golbeck	Golbeck et al. (2017)	Twitter	Harassing	Expert

**Table 2:** Comparison of studies that looked at cross-dataset (“cross-domain”) generalisation, by datasets and models used. Dataset types: H: hate speech, O: other offensive language, *: contains subtypes. Most studies carried out cross-dataset experiments, training and testing each model on all datasets. The exceptions are: Gröndahl et al. (2018) and Nejadgholi & Kiritchenko (2020) used different datasets for training and testing; Fortuna, Soler & Wanner (2020) compared datasets through class vector representations.

		Study											
Dataset name	Type	Karan & Šnajder (2018)	Gröndahl et al. (2018)	Waseem (2016)	Wiegand, Ruppenhofer & Kleinbauer (2019)	Swamy, Jamatia & Gambäck (2019)	Pamungkas & Patti (2019), Pamungkas, Basile & Patti (2020)	Arango, Prez & Poblete (2020)	Fortuna, Soler & Wanner (2020)	Caselli et al. (2020)	Nejadgholi & Kiritchenko (2020)	Glavaš, Karan & Vulić (2020)	Fortuna, Soler-Company & Wanner (2021)
Waseem	H*	✓	✓	✓	✓	✓	✓	✓	✓		✓		✓
Davidson	H,O		✓	✓		✓			✓				✓
Founta	H,O				✓	✓					✓		✓
HatEval	H*						✓	✓	✓	✓			✓
Kaggle	H,O*	✓			✓				✓				✓
Gao	H	✓										✓	
AMI	H*						✓		✓				✓
Warner	H				✓								
Zhang	H		✓										
Stromfront	H												✓
TRAC	O	✓			✓				✓			✓	✓
Wulczyn	O	✓	✓								✓	✓	
OLID	O					✓	✓			✓			✓
AbuseEval	O*									✓			
Kolhatkar	O	✓											
Razavi	O				✓								
Golbeck	O						✓						
model		SVM	LR, MLP, LSTM, CNN-GRU	MLP	FastText	BERT	SVM, LSTM, GRU, BERT	LSTM, GBDT	N/A	BERT	BERT	BERT, RoBERTa	BERT, ALBERT, fastText, SVM

### Models

First of all, **model performance had been severely over-estimated**. This includes existing “state-of-the-art” models and common baselines. Models used in the experiments ranged from neural networks—deep or shallow—to classical machine learning methods, including mixtures of both. When applied cross-dataset, all show a significant performance drop. Performance on a different dataset highlights that the test set of the same dataset does not realistically represent the distribution of unseen data.

Earlier (before 2019) state-of-the-art models often involved recurrent neural networks (Gröndahl et al., 2018).

For example, the CNN-GRU model by Zhang, Robinson & Tepper (2018) first extracts 2 to 4-gram features using convolutional layers with varying kernel sizes on word embeddings, then captures the sequence orders of these features with a gated recurrent unit (GRU) layer. This model outperformed previous models on six datasets when tested in-dataset. However, when tested cross-dataset by Gröndahl et al. (2018), the model’s performance dropped even more than an LSTM, by over 30 points in macro-averaged F1.

Similarly, Badjatiya et al. (2017)’s model was once considered state-of-the-art when trained and evaluated on *Waseem*. Their two-stage training first produces word embeddings using a Long Short-Term Memory (LSTM) network through the same hate speech classification task, based on which another Gradient-Boosted Decision Tree (GBDT) classifier was trained. Arango, Prez & Poblete (2020) showed a similar F1 drop of around 30 points when applied on *HatEval*, and discussed a crucial methodological flaw—overfitting induced by extracting features on the combination of training and test set. Gröndahl et al. (2018) also reported that they failed to reproduce Badjatiya et al. (2017)’s results. Both Gröndahl et al. (2018) and Arango, Prez & Poblete (2020) also tested a Long Short-Term Memory (LSTM) network, which had been commonly used as a strong baseline. The performance drop was similar to the above two state-of-the-art models by Zhang, Robinson & Tepper (2018) and Badjatiya et al. (2017).

Since the introduction of BERT (Devlin et al., 2019), itself and its variants have been established as the new state-of-the-art. This is seen through the comparison to other neural networks (Swamy, Jamatia & Gambäck, 2019) and on the leaderboards of shared tasks, such as Zampieri et al. (2020); Fersini, Nozza & Rosso (2020). The general approach is to fine-tune a model, which had been pre-trained on domain-general data, on a target classification dataset. Yet, BERT and its variants are no exception to the lack of generalisation, although the cross-dataset performance drop is seemingly smaller. In cross-dataset experiments with four datasets, macro-averaged F1 scores decreased by 2 to 30 points (Swamy, Jamatia & Gambäck, 2019), which is less drastic compared to earlier state-of-the-art neural networks tested in other studies (Gröndahl et al., 2018; Arango, Prez & Poblete, 2020). Pamungkas, Basile & Patti (2020) and Fortuna, Soler-Company & Wanner (2021) also found that BERT and ALBERT tended to generalise the best across the models they experimented with.

Building upon BERT, a handful of recent studies suggest that additional hate-specific knowledge from outside the fine-tuning dataset might help with generalisation. Such knowledge can come from further masked language modelling pre-training on an abusive corpus (Caselli et al., 2021), or features from a hate speech lexicon (Koufakou et al., 2020).

Other models that have been studied include traditional machine learning models, such as character n-gram Logistic Regression (Gröndahl et al., 2018), character n-gram Multi-Layer Perceptron (MLP) (Gröndahl et al., 2018; Waseem, Thorne & Bingel, 2018), Support Vector Machines (Karan & Šnajder, 2018; Fortuna, Soler-Company & Wanner, 2021; Pamungkas & Patti, 2019; Pamungkas, Basile & Patti, 2020), and shallow networks with pre-trained embeddings, e.g., MLP with Byte-Pair Encoding (BPE)-based subword embeddings (Heinzerling & Strube, 2018; Waseem, Thorne & Bingel, 2018) and FastText (Joulin et al., 2017a; Wiegand, Ruppenhofer & Kleinbauer, 2019; Fortuna, Soler-Company & Wanner, 2021).

Generally, these simpler models do not perform as good as deep neural networks, such as LSTM (Pamungkas & Patti, 2019) and especially BERT and its variants (Pamungkas, Basile & Patti, 2020; Fortuna, Soler-Company & Wanner, 2021), in- or cross-dataset. However, exceptions exist in some dataset combinations, especially when it comes to generalising. For example, n-gram Logistic Regression when comparing to LSTM (Gröndahl et al., 2018), SVM when comparing to LSTM and BERT (Pamungkas & Patti, 2019; Pamungkas, Basile & Patti, 2020), and FastText when comparing to BERT (Fortuna, Soler-Company & Wanner, 2021).

These cross-dataset studies only cover some of the more representative and/or recent hate speech detection models, but one can expect that the generalisation problem go beyond this small sample, and is far more ubiquitous in existing models than what these studies cover.

Despite the significance of the problem, systematic studies that compared a variety of models with datasets controlled are very limited (Arango, Prez & Poblete, 2020; Pamungkas & Patti, 2019; Pamungkas, Basile & Patti, 2020; Fortuna, Soler-Company & Wanner, 2021); there is also limited overlap in the datasets used between different studies (Table 2). Thus, one should be careful when drawing conclusions on the relative generalisability of models.

### Data

**Training data has a pronounced influence on generalisation**. The performance drops in models highlight the differences in the distribution of posts between datasets (Karan & Šnajder, 2018), yet some datasets are more similar to each other. Furthermore, certain attributes of a dataset could lead to more generalisable models.

**Similarity between datasets** varies, as there are groups of datasets that produce models that test much better on each other. For example, in Wiegand, Ruppenhofer & Kleinbauer (2019)’s study, FastText models (Joulin et al., 2017a) trained on three datasets *(Kaggle, Founta, Razavi)* achieved F1 scores above 70 when tested on one another, while models trained or tested on datasets outside this group achieved around 60 or less. In Swamy, Jamatia & Gambäck (2019)’s study with fine-tuned BERT models (Devlin et al., 2019), *Founta* and *OLID* produced models that performed well on each other. The source of such differences are usually traced back to search terms (Swamy, Jamatia & Gambäck, 2019), topics covered (Nejadgholi & Kiritchenko, 2020; Pamungkas, Basile & Patti, 2020), label definitions (Pamungkas & Patti, 2019; Pamungkas, Basile & Patti, 2020; Fortuna, Soler-Company & Wanner, 2021), and data source platforms (Glavaš, Karan & Vulić, 2020; Karan & Šnajder, 2018).

Another way of looking at generalisation and similarity is by comparing differences **between individual classes** across datasets (Nejadgholi & Kiritchenko, 2020; Fortuna, Soler & Wanner, 2020; Fortuna, Soler-Company & Wanner, 2021), as opposed to comparing datasets as a whole. In both Nejadgholi & Kiritchenko (2020) and Fortuna, Soler-Company & Wanner (2021)’s experiments, the best generalisation is achieved for more general labels such as “toxicity”, “offensive”, or “abusive”. Generalisation is not as good for finer-grained hate speech labels. All in all, these findings are indicative of an imbalance of the finer-grained subclasses, particularly owing to disagreements in the definition of what constitutes hate speech, which proves more difficult than defining what constitutes offensive language.

Within the hate speech labels, the relative similarity also varies. Fortuna, Soler & Wanner (2020) used averaged word embeddings (Bojanowski et al., 2017; Mikolov et al., 2018) to compute the representations of classes from different datasets, and compared classes across datasets. One of their observations is that *Davidson*’s “hate speech” is very different from *Waseem*’s “hate speech”, “racism”, “sexism”, while being relatively close to *HatEval*’s “hate speech” and *Kaggle*’s “identity hate”. This echoes with experiments that showed poor generalisation of models from *Waseem* to *HatEval* (Arango, Prez & Poblete, 2020) and between *Davidson* and *Waseem* (Waseem, Thorne & Bingel, 2018; Gröndahl et al., 2018).

In terms of what properties of a dataset lead to more generalisable models, there are frequently mentioned factors, but also inconsistency across different studies. Interactions between factors, which contribute to the inconsistency, are also reported.

The **proportion of abusive posts** in a dataset, first of all, plays a part. Swamy, Jamatia & Gambäck (2019) holds that a larger proportion of abusive posts (including hateful and offensive) leads to better generalisation to dissimilar datasets, such as *Davidson*. This is in line with Karan & Šnajder (2018)’s study where *Kumar* and *Kolhatkar* generalised best, and Waseem, Thorne & Bingel (2018)’s study where models trained on *Davidson* generalised better to *Waseem* than the other way round. In contrast, in Wiegand, Ruppenhofer & Kleinbauer (2019)’s study, the datasets with the least abusive posts generalised the best (*Kaggle* and *Founta*). Similarly, Fortuna, Soler-Company & Wanner (2021) could not confirm the impact of class proportions. Nejadgholi & Kiritchenko (2020) offered an explanation to this: there exists a trade-off between true positive and true negative rates dictated by the class proportions, which impacts the minority class performance the most but this is not always reflected in the overall F1 score.

**Biases in the samples** are also frequently mentioned. Wiegand, Ruppenhofer & Kleinbauer (2019) hold that less biased sampling approaches produce more generalisable models. This was later reproduced by Razo & Kübler (2020) and also helps explain their results with the two datasets that have the least positive cases. Similarly, Pamungkas & Patti (2019) mentioned that a wider coverage of phenomena lead to more generalisable models. So do topics that are more general rather than platform-specific (Nejadgholi & Kiritchenko, 2020).

A larger training **data size** is generally believed to produce better and more generalisable models (Halevy, Norvig & Pereira, 2009). It is mentioned as one of the two biggest factors contributing to cross-dataset performance in Karan & Šnajder (2018)’s study. Caselli et al. (2020) also found that, on *HatEval*, their dataset (*AbuseEval*) produced a model even better-performing than the one trained on *HatEval* end-to-end. They partially attributed this to a bigger data size, alongside **annotation quality**. However, the benefit of having more data is counterbalanced by data distribution differences (Karan & Šnajder, 2018), as discussed above. Moreover, its relative importance compared to other factors seems to be small, when the latter are carefully controlled (Nejadgholi & Kiritchenko, 2020; Fortuna, Soler-Company & Wanner, 2021).

### The cross-lingual case

Most of these studies only worked with English data. Yet, it is worth stressing that hate speech is a universal problem that exists in many languages, and generalisation studies focused on languages other than English are to date very sparse, despite the importance of the problem. Thus, **research on cross-lingual generalisation is still in early stages**.

One way to look at generalisation in non-English hate speech detection is applying the same cross-dataset evaluation on multiple datasets in another language. However, such studies do not yet exist. This is related to the fact that the majority of datasets are in English, which reflects linguistic and cultural unevenness in this field of research (Poletto et al., 2020; Vidgen & Derczynski, 2020).

Cross-lingual generalisation can be considered a more “extreme” type of generalisation (Arango, Prez & Poblete, 2020). The ideal case would be to be able to use data in one language for training and apply the model on data in another language, which would help address the challenge in low-resource languages. In a few studies (Pamungkas, Basile & Patti, 2020; Glavaš, Karan & Vulić, 2020; Arango, Prez & Poblete, 2020; Fortuna, Soler-Company & Wanner, 2021), language was included as a separate variable, alongside a “domain” variable independent to it, which is characterised by the source platform or the data collection method. These cross-lingual experiments are summarised in Table 3.

**Table 3:** Cross-lingual generalisation studies. All studies included English as the main language, hence only additional languages are mentioned.

Study	Data translation	Embedding and classifier models	Additional languages
Pamungkas & Patti (2019)	Training, automatic	MUSE embeddings, LSTM, “joint-learning”	Spanish, Italian, German
Pamungkas, Basile & Patti (2020)	Training, automatic	MUSE embeddings, mBERT, LSTM, SVM, “joint-learning”	Spanish, Italian
Glavaš, Karan & Vulić (2020)	Testing, manual	mBERT, XLM-R	Albanian, Croatian, German, Russian, Turkish
Arango, Prez & Poblete (2020)	Testing, automatic	MUSE embeddings, LSTM, GBDT	Spanish
Fortuna, Soler-Company & Wanner (2021)	None	mBERT	Italian, Spanish, Portuguese

Although these studies all touch on the same problem, how they evaluate cross-lingual performance differs. There are two main ways of enabling cross-lingual experiments: translating data and using multi-lingual models. These studies differ mainly by whether they perform translation on training or testing data and whether the translation is automatic or manual. Studies that use different evaluation methods also tend to look at the difficulty of the task differently. For example, Fortuna, Soler-Company & Wanner (2021) hold that multilingual generalisation per se is likely to be worse than its monolingual counterpart, while Arango, Prez & Poblete (2020) consider the two types of generalisation similar.

The factors that contribute to cross-lingual generalisation are similar to those in the monolingual setting as discussed above, with a few additional challenges:

• In terms of **models**, pre-trained multilingual word embeddings (MUSE (Conneau et al., 2017)) and language models (mBERT (Devlin et al., 2019), XLM-R (Conneau et al., 2020)) are frequently chosen as baselines. They are an intuitive and easily accessible starting point for cross-lingual experiments, but their limitations are also clear—the “curse of multilinguality” trades off single-language performance for its broad language coverage, as displayed in the results of the cross-lingual generalisation studies mentioned above (Pamungkas & Patti, 2019; Pamungkas, Basile & Patti, 2020; Glavaš, Karan & Vulić, 2020; Arango, Prez & Poblete, 2020; Fortuna, Soler-Company & Wanner, 2021) and in other tasks (Conneau et al., 2020). Similarly to the monolingual case, there are cases where traditional machine learning models outperform deep learning ones, such as SVM (Pamungkas, Basile & Patti, 2020) and GBDT (Arango, Prez & Poblete, 2020) compared to LSTM. Adding automatically translated training data alongside the original is beneficial (Pamungkas & Patti, 2019; Pamungkas, Basile & Patti, 2020).
• When it comes to the **data**, the most prominent additional factor compared to the monolingual setting is the similarity between the training (source) and testing (target) languages. For instance, Among the wide range of languages that Glavaš, Karan & Vulić (2020) have tested, the cross-lingual performance drop between English, the source language, and German, the most similar target language, was less than one third of that between English and Turkish, when using mBERT on *Wulczyn*.

Although these studies more or less consider the “language” and “domain” variables as separate, there exists evidence that the two types of generalisation interact with each other. Studies that control the language variable more carefully tend to show a smaller drop across languages—for example, by manually translating exactly the same data (Glavaš, Karan & Vulić, 2020), as opposed to using automatic translation (Pamungkas & Patti, 2019; Pamungkas, Basile & Patti, 2020; Arango, Prez & Poblete, 2020) or different language dataset from the same shared task (Pamungkas & Patti, 2019; Pamungkas, Basile & Patti, 2020; Fortuna, Soler-Company & Wanner, 2021). Furthermore, adding data from a different domain can act as a regulariser from overfitting to the training language (Glavaš, Karan & Vulić, 2020).

As more datasets emerge, we can expect more generalisation studies considering language as a parameter in the near future. For the remainder of this paper, we discuss issues that can apply to hate speech detection in any language.

## Obstacles to Generalisable Hate Speech Detection

Demonstrating the lack of generalisability is only the first step in understanding this problem. This section delves into three key factors that contribute to it: (1) presence of non-standard grammar and vocabulary, (2) paucity of and biases in datasets, and (3) implicit expressions of hate.

### Non-standard grammar and vocabulary

Hate speech detection, which is largely focused on social media, shares similar challenges to other social media tasks and has its specific ones, when it comes to the grammar and vocabulary used. Such user language style introduces challenges to generalisability at the data source, mainly by making it difficult to utilise common NLP pre-training approaches.

On social media, syntax use is generally more casual, such as the omission of punctuation (Blodgett & O’Connor, 2017). Alternative spelling and expressions are also used in dialects (Blodgett & O’Connor, 2017), to save space, and to provide emotional emphasis (Baziotis, Pelekis & Doulkeridis, 2017). Sanguinetti et al. (2020) provided extensive guidelines for studying such phenomena syntactically.

Commonly seen in hate speech, the offender adopts various approaches to evade content moderation. For example, the spelling of offensive words or phrases can be obfuscated (Nobata et al., 2016; Serrà et al., 2017), and common words such as “Skype”, “Google”, and “banana” may have a hateful meaning—sometimes known as euphemism or code words (Taylor, Peignon & Chen, 2017; Magu & Luo, 2018).

When the spelling is obfuscated, a word is considered out-of-vocabulary and thus no useful information can be given by the pre-trained models. In the case of code words, pre-trained embeddings will not reflect its context-dependent hateful meaning. At the same time, simply using identified code words for a lexicon-based detection approach will result in low precision (Davidson et al., 2017). As there are infinite ways of combining the above alternative rules of spelling, code words, and syntax, hate speech detection models struggle with these rare expressions even with the aid of pre-trained word embeddings.

In practice, this difficulty is manifested in false negatives. Qian et al. (2018) found that rare words and implicit expressions are the two main causes of false negatives; Van Aken et al. (2018) compared several models that used pre-trained word embeddings, and found that rare and unknown words were present in 30% of the false negatives of Wikipedia data and 43% of Twitter data. Others have also identified rare and unknown words as a challenge for hate speech detection (Nobata et al., 2016; Zhang & Luo, 2018). More recently, Fortuna, Soler-Company & Wanner (2021) drew a more direct line between out-of-vocabulary words and generalisation performance, by showing that the former is one of the top contributing features in a classifier for the latter. It has also been shown as an important factor in the cross-lingual case (Pamungkas, Basile & Patti, 2020).

### Existing solutions

From a domain-specific perspective, Taylor, Peignon & Chen (2017) and Magu & Luo (2018) attempted to **identify code words** for slurs used in hate communities. Both of them used keyword search as part of their sourcing of Twitter data and word embedding models to model word relationships. Taylor, Peignon & Chen (2017) identified hate communities through Twitter connections of the authors of extremist articles and hate speech keyword searches. They trained their own dependency2vec (Levy & Goldberg, 2014) and FastText (Bojanowski et al., 2017) embeddings on the hate community tweets and randomly sampled “clean” tweets, and used weighted graphs to measure similarity and relatedness of words. Strong and weak links were thus drawn from unknown words to hate speech words. In contrast, Magu & Luo (2018) collected potentially hateful tweets using a set of known code words. They then computed the cosine similarity between all words based on a word2vec model (Mikolov et al., 2013) pre-trained on news data. Code words, which have a neutral meaning in news context, were further apart from other words which fit in the hate speech context. Both Taylor, Peignon & Chen (2017) and Magu & Luo (2018) focused on the discovery of such code words and expanding relevant lexicons, but their methods could potentially complement existing hate lexicons as classifier features or for data collection.

Recently, an increasing body of research is approaching the problem by adapting character or sequence-level features to evade the challenge posed by words:

The benefit of **character-level features** has not been consistently observed. Three studies compared character-level, word-level, and hybrid (both character- and word-level) CNNs, but drew completely different conclusions. Park (2018) and Meyer & Gambäck (2019) found hybrid and character CNN to perform best respectively. Probably most surprisingly, Lee, Yoon & Jung (2018) observed that word and hybrid CNNs outperformed character CNN to similar extents, with all CNNs performing worse than character n-gram logistic regression. Small differences between these studies could have contributed to this inconsistency. More importantly, unlike the word components of the models, which were initialised with pre-trained word embeddings, the character embeddings were trained end-to-end on the very limited respective training datasets. It is thus likely that these character embeddings overfit on the training data.

In contrast, simple character n-gram logistic regression has shown results as good as sophisticated neural network models, including the above CNNs (Van Aken et al., 2018; Gao & Huang, 2017; Lee, Yoon & Jung, 2018). Indeed, models with fewer parameters are less likely to overfit. This suggests that character-level features themselves are very useful, when used appropriately. A few studies used word embeddings that were additionally enriched with subword information as part of the pre-training. For example, FastText (Bojanowski et al., 2017) models were consistently better than hybrid CNNs (Bodapati et al., 2019). In addition, a MIMICK (Pinter, Guthrie & Eisenstein, 2017)-based model displayed similar performances (Mishra, Yannakoudakis & Shutova, 2018).

The use of **sentence embeddings** partially solves the out-of-vocabulary problem by using the information of the whole post instead of individual words. Universal Sentence Encoder (Cer et al., 2018), combined with shallow classifiers, helped one team (Indurthi et al., 2019) achieve first place at the HatEval 2019 shared task (Basile et al., 2019). Sentence embeddings, especially those trained with multiple tasks, also consistently outperformed traditional word embeddings (Chen, McKeever & Delany, 2019).

**Large language models** with sub-word information have the benefits of both subword-level word embeddings and sentence embeddings. They produce the embedding of each word with its context and word form. Indeed, BERT (Devlin et al., 2019) and its variants have demonstrated top performances at hate or abusive speech detection challenges recently (Liu, Li & Zou, 2019; Mishra & Mishra, 2019).

Nonetheless, these relatively good solutions to out-of-vocabulary words (subword- and context-enriched embeddings) all face the same short-coming: they have only seen the standard English retrieved from BookCorpus and Wikipedia. NLP tools perform best when trained and applied in specific domains (Duarte, Llanso & Loup, 2018). In hate speech detection, word embeddings trained on relevant data (social media or news sites) had a clear advantage (Chen, McKeever & Delany, 2018; Vidgen et al., 2020). The domain mismatch could have similarly impaired the subword- and context-enriched models’ performances. There is little work so far on adapting them to the abusive domain to increase model generalisability so far (Caselli et al., 2021).

### Limited, biased labelled data

#### Small data size

Obstacles to generalisability also lie in dataset construction, and dataset size is the relatively most unequivocal one. When using machine learning models, especially deep learning models with millions of parameters, small dataset size can lead to overfitting and in turn harm generalisability (Goodfellow, Bengio & Courville, 2016).

It is particularly challenging to acquire labelled data for hate speech detection as knowledge or relevant training is required of the annotators. As a high-level and abstract concept, the judgement of “hate speech” is subjective, needing extra care when processing annotations. Hence, datasets are usually not big in size.

### Existing solutions

The use of **pre-trained embeddings** (discussed earlier) and parameter dropout (Srivastava et al., 2014) have been accepted as standard practice in the field of NLP to prevent over-fitting, and are common in hate speech detection as well. Nonetheless, the effectiveness of domain-general embedding models is questionable, and there has been only a limited number of studies that looked into the *relative* suitability of different pre-trained embeddings on hate speech detection tasks (Chen, McKeever & Delany, 2018; Mishra, Yannakoudakis & Shutova, 2018; Bodapati et al., 2019).

In Swamy, Jamatia & Gambäck (2019)’s study of model generalisability, **abusive language-specific pre-trained embeddings** were suggested as a possible solution to limited dataset sizes. Alatawi, Alhothali & Moria (2020) proposed White Supremacy Word2Vec (WSW2V), which was trained on one million tweets sourced through white supremacy-related hashtags and users. Compared to general word2vec (Mikolov et al., 2013) and GloVe (Pennington, Socher & Manning, 2014) models trained on news, Wikipedia, and Twitter data, WSW2V captured meaning more suitable in the hate speech context –e.g., ambiguous words like “race” and “black” have higher similarity to words related to ethnicity than sports or colours. Nonetheless, their WSW2V-based LSTM model did not consistently outperform Twitter GloVe-based LSTM model or BERT (Devlin et al., 2019). They did not consider cross-dataset testing for generalisablity, either.

The pre-training for BERT (and its variants) is both data and computationally-heavy, which limits the feasibility of training the hate speech equivalent of BERT from scratch. A reasonable compromise to that is performing further Masked Language-Modelling pre-training before the fine-tuning stage. By further pre-training RoBERTa (Liu et al., 2019), Wiedemann, Yimam & Biemann (2020) achieved first place at the Offenseval 2020 shared task (Zampieri et al., 2020). Caselli et al. (2021) pre-trained BERT further on a larger-scale dataset of banned abusive subreddits and observed improvement over standard BERT on three Twitter datasets (*OLID, AbuseEval, HatEval*), in-dataset for all cases and cross-dataset for most cases. Both studies show that abusive language-specific pre-training, built upon generic pre-training, can be beneficial for both in-dataset performance and cross-dataset generalisation. The main downside is that the improvement gains, ranging from less than 1% to 4% in macro F1, seem disproportionate to the computational cost—Wiedemann, Yimam & Biemann (2020) only did the training on a small sample due to hardware limitations; it took Caselli et al. (2021) 18 days to complete 2 million training steps on one Nvidia V100 GPU. There also exists a trade-off between precision and recall for the positive class due to the domain shift (Caselli et al., 2021).

Research on **transfer learning from other tasks**, such as sentiment analysis, also lacks consistency. Uban & Dinu (2019) pre-trained a classification model on a large sentiment dataset (https://help.sentiment140.com/), and performed transfer learning on the *OLID* and *Kumar* datasets. They took pre-training further than the embedding layer, comparing word2vec (Mikolov et al., 2013) to sentiment embeddings and entire-model transfer learning. Entire-model transfer learning was found to be always better than using the baseline word2vec (Mikolov et al., 2013) model, but the transfer learning performances with only the sentiment embeddings were not consistent.

More recently, Cao, Lee & Hoang (2020) also trained sentiment embeddings through classification as part of their proposed model. The main differences are: the training data was much smaller, containing only *Davidson* and *Founta* datasets; the sentiment labels were produced by VADER (Gilbert & Hutto, 2014); their model was deeper and used general word embeddings (Mikolov et al., 2013; Pennington, Socher & Manning, 2014; Wieting et al., 2015) and topic representation computed through Latent Dirichlet Allocation (LDA) (Blei, Ng & Jordan, 2003) in parallel. Through ablation studies, they showed that sentiment embeddings were beneficial for both *Davidson* and *Founta* datasets.

Use of existing knowledge from a more mature research field like that of sentiment analysis has the potential to be used to jumpstart the relatively newer field of hate speech detection. It also offers a compromise between hate speech models, which might not be generalisable enough, and completely domain-general models, which lack knowledge specific to hate speech detection. Nonetheless, more investigation into the conditions in which transfer learning works best to increase generalisability in particular still needs to be done.

#### Sampling bias

In addition to a limited size, datasets are also prone to biases. Non-random sampling and subjective annotations introduce individual biases, and the different sampling and annotation processes across datasets further increase the difficulty of training models that can generalise across heterogeneous data.

Hate speech and, more generally, offensive language generally represent less than 3% of social media content (Zampieri et al., 2019b; Founta et al., 2018). To alleviate the effect of scarce positive cases on model training, all existing social media hate speech or offensive content datasets used boosted (or focused) sampling with simple heuristics.

Table 4 compares the **sampling methods** of hate speech datasets studied the most in cross-dataset generalisation. Consistently, keyword search and identifying potential hateful users are the most common methods. However, what is used as the keywords (slurs, neutral words, profanity, hashtags), which users are included (any user from keyword search, identified haters), and the use of other sampling methods (identifying victims, sentiment classification) all vary a lot.

**Table 4:** Boosted sampling methods of the most commonly studied hate speech datasets (Waseem & Hovy, 2016; Davidson et al., 2017; Founta et al., 2018; Basile et al., 2019). Description as appeared in the publications.

Dataset	Keywords	Haters	Other
Waseem	“Common slurs and terms used pertaining to religious, sexual, gender, and ethnic minorities”	“A small number of prolific users”	N/A
Davidson	HateBase (https://www.hatebase.org/)	“Each user from lexicon search”	N/A
Founta	HateBase, NoSwearing (https://www.noswearing.com/dictionary/)	N/A	Negative sentiment
HatEval	“Neutral keywords and derogatory words against the targets, highly polarized hashtags”	“Identified haters”	“Potential victims of hate accounts”

**Notes.**

N/A no relevant descriptions found

**Table 5:** Annotation guidelines of the most commonly studied hate speech datasets.

Dataset	Action	Target	Clarifications
Waseem	Attacks, seeks to silence, criticises, *negatively stereotypes, promotes hate speech or violent crime, blatantly misrepresents truth or seeks to distort views on*, uses a sexist or racial slur, defends xenophobia or sexism	A minority	(Inclusion) Contains a screen name that is offensive, as per the previous criteria, the tweet is ambiguous (at best), and the tweet is on a topic that satisfies any of the above criteria
Davidson	*Express hatred towards*, humiliate, insult^*^	A group or members of the group	(Exclusion) Think not just about the words appearing in a given tweet but about the context in which they were used; the presence of a particular word, however offensive, did not necessarily indicate a tweet is hate speech
Founta	*Express hatred towards*, humiliate, insult	Individual or group, on the basis of attributes such as race, religion, ethnic origin, sexual orientation, disability, or gender	N/A
HatEval	*Spread, incite, promote, justify hatred or violence towards*, dehumanizing, hurting or intimidating^**^	Women or immigrants	(Exclusion) Hate speech against other targets, offensive language, blasphemy, historical denial, overt incitement to terrorism, offense towards public servants and police officers, defamation

**Notes.**

Original wording from the publications or supplementary materials; action verbs grouped for easier comparison:

underlined directly attack or attempt to hurt, *italic* promote hate towards. N/A no relevant descriptions found

* Davidson et al. (2017) also gave annotators “a paragraph explaining it (the definition) in further detail”, which was not provided in their publication.

** Basile et al. (2019) also gave annotators some examples in their introduction of the task (rather than the main guidelines, thus not included).

Moreover, different studies are based on varying definitions of “hate speech”, as seen in different **annotation guidelines** (Table 5). Despite all covering the same two main aspects (directly attack or promote hate towards), datasets vary by their wording, what they consider a target (any group, minority groups, specific minority groups), and their clarifications on edge cases. *Davidson* and *HatEval* both distinguished “hate speech” from “offensive language”, while “uses a sexist or racist slur” is in *Waseem*’s guidelines to mark a case positive of hate, blurring the boundary of offensive and hateful. Additionally, as both *HatEval* and *Waseem* specified the types of hate (towards women and immigrants; racism and sexism), hate speech that fell outside of these specific types were not included in the positive classes, while *Founta* and *Davidson* included any type of hate speech. Guidelines also differ in how detailed they are: Apart from *Founta*, all other datasets started the annotation process with sets of labels pre-defined by the authors, among which *Waseem* gave the most specific description of actions. In contrast, *Founta* only provided annotators with short conceptual definitions of a range of possible labels, allowing more freedom for a first exploratory round of annotation. After that, labels were finalised, and another round of annotation was carried out. As a result, the labelling reflects how the general public, without much domain knowledge or extensive training, would classify offensive language. For example, the “abusive” and “offensive” classes were so similar that they were merged in the second stage. However, as discussed above, they differ by whether intentionality is present (Caselli et al., 2020). Such different annotation and labelling criteria result in essentially different tasks and different training objectives, despite their data having a lot in common.

As a result of the varying and sampling methods, definitions, and annotation schemes, what current models can learn on one dataset is specific to the examples in that dataset and the task defined by the dataset, limiting the models’ ability to generalise to new data. One type of possible resulting bias is **author bias**. For example, 65% of the hate speech in the *Waseem* dataset was produced by merely two users, and their tweets exist in both the training and the test set. Models trained on such data thus overfit to these users’ language styles. This overfitting to authors was proven in two state-of-the-art models (Badjatiya et al., 2017; Agrawal & Awekar, 2018; Arango, Prez & Poblete, 2020). **Topic bias** is another concern. With words such as “football” and “announcer” among the ones with the highest Pointwise Mutual Information (PMI) with hate speech posts, a topic bias towards sports was demonstrated in the *Waseem* dataset (Wiegand, Ruppenhofer & Kleinbauer, 2019). Such biases can also be measured through the semantic similarity between keywords used for building the datasets and topics present in the dataset (Ousidhoum, Song & Yeung, 2020).

### Existing solutions

A few recent studies have attempted to go beyond one dataset when training a model.

Waseem, Thorne & Bingel (2018) used **multitask training** (Caruana, 1997) with hard parameter sharing up to the final classification components, which were each tuned to one hate speech dataset. The shared shallower layers, intuitively, extract features useful for both datasets, with the two classification tasks as regularisation against overfitting to either one. Their multitask-trained models matched the performances of models trained end-to-end to single datasets and had clear advantage over simple dataset concatenation, whilst allowing generalisation to another dataset. Karan & Šnajder (2018) presented a similar study. Frustratingly Easy **Domain Adaptation** (Daumé III, 2007) had similar beneficial effects but was much simpler and more efficient. These two studies showed the potential of combining datasets to increase generalisability, but further investigation into this approach is lacking.

#### Representation bias

A different kind of bias is representation bias. To put simply, models trained on “norms” will fail to generalise to data far from the “norms”. This also harms model generalisability in a much broader sense, mainly through application practicality.

Natural language is a proxy of human behaviour, thus the biases of our society are reflected in the datasets and models we build. With increasing real-life applications of NLP systems, these biases can be translated into wider social impacts (Hovy & Spruit, 2016). Minority groups are underrepresented in available data and/or data annotators, thus causing biases against them when models are trained from this data. This phenomenon is also seen in audio transcribing (Tatman, 2017), sentiment analysis (Kiritchenko & Mohammad, 2018), etc.

Hate speech detection models not only have higher tendency to classify African-American English posts as offensive or hate than “white” English (Davidson, Bhattacharya & Weber, 2019), but also more often predict false negatives on “white” than African-American English (Sap et al., 2020). Certain words and phrases, including neutral identity terms such as “gay” (Dixon et al., 2018) and “woman” (Park, Shin & Fung, 2018) can also easily lead to a false positive judgement. Moreover, just like biases in real life, racial, gender, and party identification biases in hate speech datasets were found to be intersectional (Kim et al., 2020).

The prevalence of such biases mean that existing hate speech detection models are likely to struggle at generalising to unseen data that contain expressions related to these demographic groups. Furthermore, compared to the other types of biases mentioned above, they do more harm to the practical value of the automatic hate speech detection models. These biases may cause automatic models to amplify the harm against minority groups instead of mitigating such harm as intended (Davidson, Bhattacharya & Weber, 2019). For example, with higher false positive rates for minority groups, their already under-represented voice will be more often falsely censored.

### Existing solutions

Systematic studies of representation biases and their mitigation are relatively recent. Since Dixon et al. (2018) first quantified unintended biases in abusive language detection on the *Wulczyn* dataset using a synthetic test set, an increasing number of studies have been carried out on hate speech and other offensive language. These attempts to address biases against minority social groups differ by how they measure biases and their approaches to mitigate them.

Similar to Dixon et al. (2018), a number of studies measured bias as certain words and phrases being associated with the hateful or offensive class, which were mostly identity phrases. Attempts to mitigate biases identified this way focus on decoupling this association between features and classes. Model performance on a **synthetic test set** with classes and identity terms balanced, compared to the original test data, were used a measure for model bias. Well-known identity terms and synonyms are usually used as starting points (Dixon et al., 2018; Park, Shin & Fung, 2018; Nozza, Volpetti & Fersini, 2019). Alternatively, bias-prone terms could be identified through looking at skewed distributions within a specific dataset (Badjatiya, Gupta & Varma, 2019; Mozafari, Farahbakhsh & Crespi, 2020b).

A few studies measured biases across directly **predicted language styles or demographic attributes** of authors. Davidson, Bhattacharya & Weber (2019) and Kim et al. (2020) both tested their hate speech detection models on Blodgett, Green & OConnor (2016)’s distantly supervised dataset of African-American vs white-aligned English tweets, revealing higher tendencies of labelling an African-American-aligned tweet offensive or hateful. Kim et al. (2020) further extended this observation to gender and party identification. As the testing datasets do not have hateful or offensive ground truth labels, one caveat is that, using this as a metric of model bias assumes that all language styles have equal chances of being hateful or offensive, which might not be true.

Huang et al. (2020) approached author demographics from a different angle, and instead predicted author demographics on available hate speech datasets using user profile descriptions, names, and photos. They built and released a multilingual corpus for model bias evaluation. Although now with ground truth hate speech labels, this introduces additional possible bias existing in the tools they used into the bias evaluation process. For example, they used a computer vision API on the profile pictures to predict race, age, and gender, which displayed racial and gender biases (Buolamwini & Gebru, 2018).

One mitigation approach that stemmed from the first approach of measuring biases is “debiasing” training data through **data augmentation**. Dixon et al. (2018) retrieved non-toxic examples containing a range of identity terms following a template, which were added to *Wulczyn*. Following a similar logic, Park, Shin & Fung (2018) created examples containing the counterpart of gendered terms found in the data to address gender bias in the *Waseem* and *Founta* datasets. Badjatiya, Gupta & Varma (2019) extended this word replacement method by experimenting with various strategies including named entity tags, part of speech tags, hypernyms, and similar words from word embeddings, which were then applied on the *Wulczyn* and *Davidson* datasets.

Less biased **external corpora and pre-trained models** could also be used. To reduce gender bias, Park, Shin & Fung (2018) also compared pre-trained debiased word embeddings (Bolukbasi et al., 2016) and transfer learning from a larger, less biased corpus. Similarly, Nozza, Volpetti & Fersini (2019) added samples from the *Waseem* dataset to their training dataset (*AMI*), to keep classes and gender identity terms balanced.

From the perspective of model training, biases could also be understood through **model explanation**, and “debiasing” could be accordingly integrated into the **model training objective**. Based on 2-grams’ Local Mutual Information with a label, Mozafari, Farahbakhsh & Crespi (2020b) gave each training example in the *Davidson* and *Waseem* datasets a positive weight, producing a new weighted loss function to optimise. Kennedy et al. (2020) built upon a recent study of post-hoc BERT feature importance (Jin et al., 2020). A regularisation term to encourage the importance of a set of identity terms to be close to zero was added to the loss function. This changed the ranks of importance beyond the curated set of identity terms in the final model trained on two datasets (de Gibert et al., 2018; Kennedy et al., 2018), with that of most identity terms decreasing, and some aggressive words increasing, such as “destroys”, “poisoned”. Vaidya, Mai & Ning (2019) used a similar multitask learning framework to Waseem, Thorne & Bingel (2018) on *Kaggle*, but with the classification of author’s identity as the auxiliary task to mitigate the confusion between identity keywords and hateful reference. Similarly, Xia, Field & Tsvetkov (2020) incorporated the prediction of African-American English dialect in their loss term, but this was done after an initial pre-training of the hate speech classification alone.

There is little consensus in how bias and the effect of bias mitigation should be measured, with different studies adopting varying “debiased” metrics, including Error Rate Equality Difference (Dixon et al., 2018; Park, Shin & Fung, 2018; Nozza, Volpetti & Fersini, 2019), pinned AUC Equality Difference (Dixon et al., 2018; Badjatiya, Gupta & Varma, 2019), Pinned Bias (Badjatiya, Gupta & Varma, 2019), synthetic test set AUC (Park, Shin & Fung, 2018), and weighted average of subgroup AUCs (Nozza, Volpetti & Fersini, 2019; Vaidya, Mai & Ning, 2019). More importantly, such metrics are all defined based on how the subgroups are defined –which datasets are used, which social groups are compared, which keywords or predictive models are chosen to categorise those groups. As a consequence, although such metrics provide quantitative comparison between different mitigation strategies within a study, the results are hard to compare horizontally. Nonetheless, a common pattern is found across the studies: the standard metric, such as raw F1 or AUC, and the “debiased” metrics seldom improve at the same time. This raises the question on the relative importance that should be put on “debiased” metrics and widely accepted raw metrics: how much practical value do such debiased metrics have if they contradict raw metrics? Or do we need to rethink the widely accepted AUC and F1 scores on benchmark datasets because they do not reflect the toll on minority groups?

In comparison, Sap et al. (2019) proposed to address the biases of human annotators during dataset building, rather than debiasing already annotated data or regularising models. By including each tweet’s dialect and providing **extra annotation instructions** to think of tweet dialect as a proxy of the author’s ethnic identity, they managed to significantly reduce the likelihood of the largely white annotator group (75%) to rate an African-American English tweet offensive to anyone or to themselves. This approach bears similarity to Vaidya, Mai & Ning (2019)’s, which also sought to distinguish identity judgement from offensiveness spotting, although in automatic models. Although on a small scale, this study demonstrated that more care can be put into annotator instructions than existing datasets have.

### Hate expression can be implicit

Implicit expressions are an obstacle to generalisability that comes from the nature of hate speech, and is arguably the trickiest to address. Compared to explicity, which is more transferable between datasets (Nejadgholi & Kiritchenko, 2020), implicity poses challenges to generalisation through interacting with the aforementioned two obstacles: in implicit expressions, there are fewer lexical features to be learnt, and limited, biased data further magnify the challenge of learning generalisable features; implicit hate expressions diverge from standard language use even further than social media or explicit hate speech.

Slurs and profanity are common in hate speech. This is partly why keywords are widely used as a proxy to identify hate speech in existing datasets. However, hate can also be expressed through stereotypes (Sap et al., 2020), sarcasm, irony, humour, and metaphor (Mishra, Yannakoudakis & Shutova, 2019; Vidgen et al., 2019). For example, a post that reads “Hey Brianne - get in the kitchen and make me a samich. Chop Chop” (Gao & Huang, 2017) *directly attacks* a woman *based on* her female *identity* using stereotypes, fufilling the definition of hate speech without any distinctive keyword.

Implicit hate speech conveys the same desire to distance such social groups as explicit hate speech (Alorainy et al., 2019) and are no less harmful (Breitfeller et al., 2019). Implicit expressions are the most commonly mentioned cause of false negatives in error analysis (Zhang & Luo, 2018; Qian et al., 2018; Basile et al., 2019; Mozafari, Farahbakhsh & Crespi, 2020a). Inability to detect nuanced, implicit expressions of hate means the models do not go beyond lexical features and cannot capture the underlying hateful intent, let alone generalise to hate speech cases where there are no recurring hate-related words and phrases. Because of the reliance on lexical features, automatic detection models fall far short of human’s ability to detect hate and are thus far from being applicable in the real world as a moderation tool (Duarte, Llanso & Loup, 2018).

It has been proposed that abusive language should be systematically classified into explicit and implicit, as well as generalised and directed (Waseem et al., 2017). Several subsequent studies have also identified nuanced, implicit expression as a particularly important challenge in hate speech detection for future research to address (Van Aken et al., 2018; Duarte, Llanso & Loup, 2018; Swamy, Jamatia & Gambäck, 2019). It is especially necessary for explainability (Mishra, Yannakoudakis & Shutova, 2019). Despite the wide recognition of the problem, there has been much fewer attempts at addressing it.

### Existing solutions

Implicit cases of hate speech are hard to identify because they can be understood only within their specific context or with the help of relevant real-world knowledge such as stereotypes. Some have thus **included context in datasets**. For example, Gao & Huang (2017) included the original news articles as the context of the comments. de Gibert et al. (2018)’s hate speech forum dataset organised sentences in the same post together, and has a “relation” label separate from “hate”/“no hate” to set apart cases which can only be correctly understood with its neighbours.

Offensive or abusive language datasets that include implicitness in annotation schemes have appeared only recently. The *AbuseEval* dataset (Caselli et al., 2020) is so far the only **dataset with a standalone “implicit” label**. They re-annotated the *OLID* dataset (Zampieri et al., 2019a), splitting the offensive class into implicitly abusive, explicitly abusive, and non-abusive. Their dataset thus offered a clearer distinction between abusiveness and offensiveness, and between implicit and explicit abuse. Sap et al. (2020) asked annotators to explicitly **paraphrase the implied statements** of intentionally offensive posts. The task defined by this dataset is thus very different from previously existing ones—it is a sequence-to-sequence task to generate implied statements on top of the classification task to identify hateful intent.

Both of their experiments reveal that predicting implicit abuse or biases remains a major challenge. Sap et al. (2020)’s model tended to output the most generic bias of each social group, rather than the implied bias in each post. Caselli et al. (2020)’s best model achieved only a precision of around .234 and a recall of 0.098 for the implicit class, in contrast to .864 and .936 for non-abusive and .640 and .509 for explicit.

To the best of our knowledge, so far there has only been one attempt at annotating the implicitness of hate speech specifically. Alatawi, Alhothali & Moria (2020) crowd-sourced annotation on a small set of tweets collected through white supremacist hashtags and user names, dividing them into implicit white supremacism, explicit white supremacism, other hate, and neutral. Unfortunately, the inter-annotator agreement was so low (0.11 Cohen’s kappa (Cohen, 1960)) that they reduced the labels into binary (hateful vs non-hateful) in the end. The main disagreements are between neutral and implicit labels. Compared to Sap et al. (2020) and Caselli et al. (2020)’s studies, their result highlights the difficulty of annotating implicit hate speech and, more fundamentally, the perception of hate speech largely depends on the reader, as posited by Waseem (2016).

Fewer studies proposed **model design motivated by implicit hate speech**. Gao, Kuppersmith & Huang (2017) designed a novel two-path model, aiming to capture both explicit hate speech with a “slur learner” path and implicit hate speech with an LSTM path. However, it is doubtful whether the LSTM path really learns to identify implicit hate speech, as it is also trained on hate speech cases acquired through initial slur-matching and the slur learner.

Targeting specific types of implicit hate speech seems more effective. Alorainy et al. (2019) developed a feature set using dependency trees, part-of-speech tags, and pronouns, to capture the us vs them sentiment in implicit hate speech. This improved classification performance on a range of classifiers including CNN-GRU and LSTM. The main shortcoming is that the performance gain was relative to unprocessed training data, so it is not clear how effective this feature set is compared to common pre-processing methods.

## Discussion

While cross-dataset testing can be a useful tool for measuring generalisability, it is important not to reduce the study of generalisability in hate speech detection to cross-dataset performance or “debiased” metrics. Ultimately, we want generalisability to the real world. Why we are developing these models and datasets, how we intend to use them, and what potential impacts they may have on the users and the wider society are all worth keeping in mind. While mathematical metrics offer quantification, our focus should always be on what we plan to address and its context. Furthermore, hate speech datasets and models should be representative of what hate speech is with no prioritising of any facets of it (Swamy, Jamatia & Gambäck, 2019), and should not discriminate against minority groups that they are intended to protect (Davidson, Bhattacharya & Weber , 2019).

Hate speech detection as a sub-field of NLP is rather new. Despite the help of established NLP methods, achieving consensus in the formulation of the problem is still ongoing work—whether it is binary, multi-class, hierarchical, how to source representative data, what metadata should be included, and where we draw the line between offensive and hateful content. Thus, no existing dataset qualifies as a “benchmark dataset” yet (Swamy, Jamatia & Gambäck, 2019). In the near future, it is likely that new datasets will continue to emerge and shape our understanding of how to study hate speech computationally. Thus, while it is important to try to solve the problems defined by existing datasets, more emphasis should be put on generalisability.

### Future research

Generalisability is a complex problem concerning every aspect of hate speech detection—dataset building, model training and evaluation, and application. Thus, obstacles to generalisabile hate speech detection are largely intertwined.

In the “obstacles” section above, we analysed the problem of generalisability and discussed existing research, organised by obstacles and their causes. Here, we suggest what can practically be done moving forward, from the specific perspectives of dataset and models, as well as other general challenges. These suggestions vary by problem complexity and generality. Nonetheless, they are all, in our opinion, critical things to keep in mind for any researcher working on hate speech detection to evaluate and improve generalisability.

#### Datasets

##### Clear label definitions.

Unclear and different definitions surrounding hate speech lead to inconsistencies in the literature and create sampling and annotation biases and disparity between datasets, which in turn harm the generalisability of models trained on such data. Thus, a prerequisite is to have clear label definitions.

Hate speech should be separated from other types of offensive language (Davidson et al., 2017; Founta et al., 2018), and abusive language from offensive language (Caselli et al., 2020). In addition to this, to address the ambiguity between types of abusive language, future datasets can cover a wider spectrum of abusive language such as personal attacks, trolling, and cyberbullying. This could be done either in a hierarchical manner like what Basile et al. (2019) and Kumar et al. (2018b) did with subtypes of hate speech and aggression respectively, or in a multi-label manner, as there might be cases where more than one can apply, as seen in Waseem & Hovy (2016)’s racism and sexism labels. At the same time, the definitions of labels should have as little overlap as possible.

### Annotation quality.

Related to clear label definitions, ensuring annotation quality would help improve generalisation by reducing the gaps between datasets and between annotations within each dataset. Guidelines range from brief descriptions of each class to long paragraphs of definitions and examples (Table 5). Yet, only about two thirds of the existing datasets report inter-anntotator agreement rates (Poletto et al., 2020). There exists a trade-off between creating a larger dataset with the help of external workers and having high-quality annotations that reflect a precise and informed understanding of hate speech. High-quality, expert-produced annotations can help produce better models (Caselli et al., 2020). At the same time, extra guidelines were shown to be effective in addressing some of the biases in crowd-sourced annotations (Sap et al., 2019). Future research can look into what type of, and how much, training or instruction is required to match the annotations of crowdworkers and experts.

### Understanding perception.

With annotation quality, another very different approach can be taken—understanding why the perception of hate diverges across annotators. This can not only improve generalisability through addressing disparity in annotations, but also help evaluate potential representation biases and disentangle implicit expressions of hate.

While clear definitions and guidelines are worth pursuing, how each individual perceives hate speech is bound to be different depending on their background (Waseem, 2016). Thus, annotator disagreement will be inevitable even with the same guidelines and training. Instead of aggregating labels into a gold standard, an alternative way of looking at such disagreement is that it reflects an actual divergence of opinions and are all valid (Basile, 2020).

More research can be done to understand why and when disagreement arises, quantitatively or qualitatively. This can be done through building datasets with annotator attributes and their judgements. Existing datasets mostly reported the number of annotators and whether they are crowdworkers, but seldom the demographics of annotators. Furthermore, within the range of “expert” annotators, there are also many possibilities, such as the authors of the papers (de Gibert et al., 2018; Mandl et al., 2019), experts in linguistics (Kumar et al., 2018a), activists (Waseem, 2016; Waseem & Hovy, 2016), experts in politics (Vidgen et al., 2020). By training models on different sets of annotations, unintended biases in models can also be better understood. Annotating implicit hate speech is especially challenging (Alatawi, Alhothali & Moria, 2020). Through improved understanding of hate speech perception, an implicit hate speech dataset could be made possible.

### Drawing representative samples.

Before the annotation process, sampling approaches can introduce bias into the dataset and affect the proportion of implicit cases, both affecting the practical value of a detection model. Drawing more representative samples can help with generalisation through alleviating these two problems.

Abusive content represent less than 3% of social media (Zampieri et al., 2019b; Founta et al., 2018), so datasets use simple heuristics to boost the proportion of the positive label. It is a better approach to start with an initial sample and then apply boosting techniques to increase the proportion of abusive posts, compared to drawing a filtered sample using offensive keywords from the beginning (Wiegand, Ruppenhofer & Kleinbauer, 2019; Razo & Kübler, 2020). Boosting techniques can also be improved, by shifting away from keywords towards other less lexical proxies of possible hate, to reduce the emphasis on explicit hate in the dataset. Future datasets should also actively address different types of possible biases, such as regularising each user’s contribution to one dataset, analysis of the topics present in the dataset, limiting the association between certain terms or language styles and a label. It will also help to measure sampling bias quantitatively (Ousidhoum, Song & Yeung, 2020).

#### Models

##### Reducing overfitting.

Overfitting harms model generalisability in any task, but the small and biased hate speech datasets magnify this problem. In addition to the dataset building process, it can be addressed through reducing model overfitting.

Overfitting can be reduced through training on more than one dataset (Waseem, Thorne & Bingel, 2018; Karan & Šnajder, 2018) or transfer learning from a larger dataset (Uban & Dinu, 2019; Alatawi, Alhothali & Moria, 2020) and/or a closely related task, such as sentiment analysis (Uban & Dinu, 2019; Cao, Lee & Hoang, 2020), yet synthesis in the literature is lacking. More work can be done on comparing different training approaches, and what characteristics of the datasets interact with the effectiveness. For example, when performing transfer learning, the trade-off between domain-specificity and dataset size and representativeness is worth investigating.

Reducing the reliance on lexical features can also help alleviate overfitting to the training dataset. Domain knowledge such as linguistic patterns and underlying sentiment of hate speech can inform model design, feature extraction or preprocessing (Alorainy et al., 2019). Future studies can look into how features of different nature can be effectively combined.

#### Debiasing models.

Unintended representation biases threaten the practicality of applying automatic hate speech detection on unseen real-world data. Model debiasing can be carried out in conjunction with the improvement and understanding of data collection and annotation.

A range of approaches could be used to make the model less biased against certain terms or language styles, from the perspectives of training data or objective. Each study shows that their approach takes some effect, yet comparison across studies is still difficult. More systematic comparisons between debiasing approaches would be helpful. This can be done by applying a range of existing approaches on a number of datasets, with a set of consistent definitions of attributes. There could also be an interaction between debiasing approaches and the types of biases. When experimenting with “debiasing”, it is important to always stay critical of any metrics used.

#### Model application and impact.

Also related to real-world application, extra care needs to be taken with model evaluation, when addressing any of the obstacles mentioned above.

To realistically evaluate model performance, dataset-wise mathematical metrics like F1/AUC should not be the only measurement. It is also important to evaluate models also on datasets not seen during training (Wiegand, Ruppenhofer & Kleinbauer, 2019), and carry out in-depth error analysis relevant to any specific challenge that the model claims to address. Evaluation methods that are aware of different possible perceptions of hate are also desirable (Basile, 2020).

Furthermore, machine learning models should be considered as part of a sociotechnical system, instead of an algorithm which only exists in relation to the input and outcomes (Selbst et al., 2019). Thus, more future work can be put into studying hate speech detection models in a wider context of application. For example, can automatic models practically aid human moderators in content moderation? In that case, how can human moderators make use of the outputs or post-hoc feature analysis(e.g., Kennedy et al. (2020)) most effectively? Would that introduce more bias or reduce bias in content moderation? Would such effects differ across different hate expressions? What would the impact be on the users of the platform? To answer these questions, interdisciplinary collaboration is needed.

#### Other general challenges

Finally, in addition to the specific challenges regarding data and models mentioned above, these general efforts should be made in parallel:

• **Open-sourcing**. Experimental studies on generalisation require access to a variety of resources, data and models as a prerequisite. Furthermore, it is only with detailed annotation guidelines and model source code made public that detailed inspection into factors that affect generalisability can be enabled. Even without a focus on generalisation per se, easier access to evaluation data and models to compare to can help shift hate speech detection research, as a whole, towards more generalisable outputs. Thus, a joint effort on open-sourcing should be made.
• **Multilingual research**. English has a disproportionate representation in available hate speech data and existing hate speech detection research. The ubiquity of hate speech in any language and culture calls for more work on lower-resource languages in hate speech research. So far, all generalisation studies that mentioned language consider it as a detection for generalisation. Such an approach can help address the challenge the scarcity of non-English data, if, for example, models trained on English annotated data only can work well on another language. Cross-lingual generalisation is thus practically valuable. On the other hand, there exists a limit to such an “extreme” type of generalisation, determined by language and culture dissimilarity and varying social events. Thus, future contribution to cross-lingual generalisation can be two-folds: increasing cross-lingual performance through model and dataset development, probing the limit of cross-lingual performance through in-depth analysis.

## Conclusion

Existing hate speech detection models generalise poorly on new, unseen datasets. Cross-dataset testing is a useful tool to more realistically evaluate model generalisation performance, but the problem of generalisability does not stop there. Reasons why generalisable hate speech detection is hard come from limits of existing NLP methods, dataset building, and the nature of online hate speech, and are often intertwined. The behaviour of social media users and especially haters poses extra challenge to established NLP methods. Small datasets make deep learning models prone to overfitting, and biases in datasets transfer to models. While some biases come from different sampling methods or definitions, others merely reflect long-standing biases in our society. Hate speech evolves with time and context, and thus has a lot of variation in expression. Existing attempts to address these challenges span across adapting state-of-the-art in other NLP tasks, refining data collection and annotation, and drawing inspirations from domain knowledge of hate speech. More work can be done in these directions to increase generalisability in two main directions: data and models. At the same time, wider context and impact should be carefully considered. Open-sourcing and multilingual research are also important.

## Additional Information and Declarations

### Data Availability

The following information was supplied regarding data availability: The paper is a literature review that did not involve raw data or code.
